# Idiopathic Hypereosinophilic Syndrome Presenting With Embolic Stroke

**DOI:** 10.7759/cureus.19307

**Published:** 2021-11-06

**Authors:** Magda S Silva, Carina Ramalho, Francelino Ferreira, Inês Maia, Anneke Joosten

**Affiliations:** 1 Internal Medicine Department, Centro Hospitalar Barreiro Montijo, Barreiro, PRT

**Keywords:** hypereosinophilic syndrome, stroke, thrombosis, aorta, idiopathic

## Abstract

Hypereosinophilic syndrome is a rare condition characterized by eosinophilia associated with organ damage, most commonly affecting the skin, lung, gastrointestinal, cardiovascular and central nervous system. The idiopathic form is characterized by the absence of other conditions associated with hypereosinophilia such as allergies, infectious, hematological, immunological, endocrine or neoplasm diseases. The authors present a clinical case of a 70-year-old man with no relevant history, who went to the emergency department for neurological deficits and nonspecific chest pain. Laboratory tests revealed marked eosinophilia, elevation of cardiac enzymes with normal electrocardiogram. Computed tomography of the head showed multiple bilateral ischemic lesions. Upon further investigation for the cerebrovascular disease, transesophageal echocardiogram showed a thrombus at the aortic arch, as a probable embolic source. Despite anticoagulant therapy and corticosteroids, the patient's status deteriorated, with multiple successive ischemic strokes and worsening neurological deficits. After a thorough investigation, the diagnosis of idiopathic eosinophilia was established.

## Introduction

Hypereosinophilic syndrome (HES) is a rare entity characterized by eosinophilia associated with organ damage, most frequently affecting the skin, lungs, central nervous systems, gastrointestinal and cardiovascular systems. The idiopathic form is defined by the absence of other entities associated with hypereosinophilia. The clinical spectrum is highly variable and depends on the organs infiltrated by eosinophils, which cause inflammation, fibrosis, vasculitis and thrombosis. It is hypothesised that the neurological symptoms result from direct effects caused by eosinophils, but can also be secondary to thromboembolic phenomena, a serious complication of HES as we reported in the clinical case.

## Case presentation

**Figure 1:**
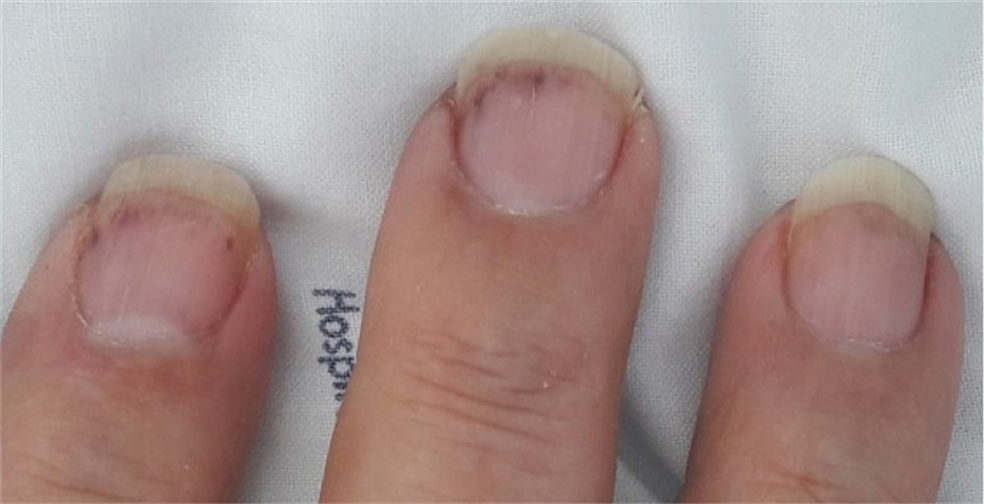
Linear subungual hemorrhages (splinter hemorrhage).

**Figure 2:**
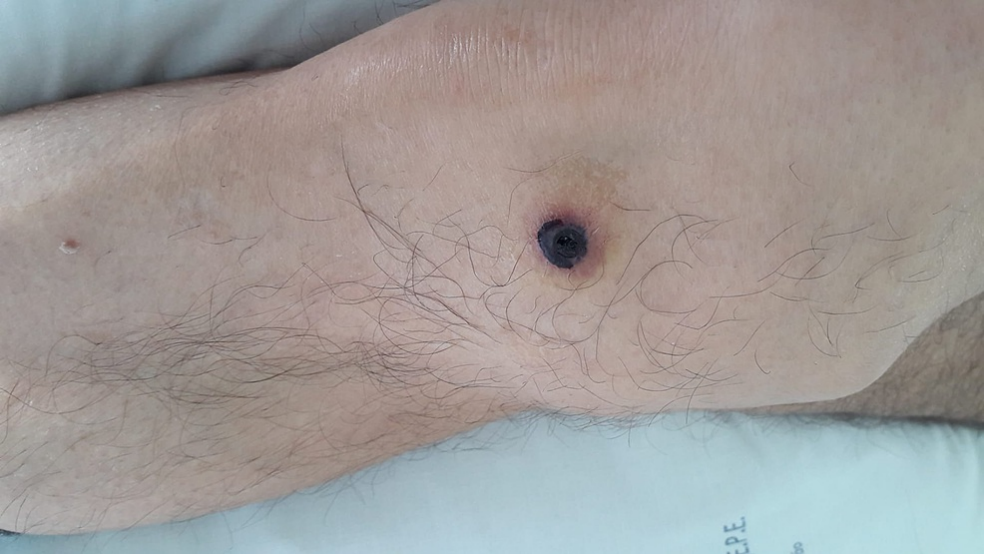
A lesion with necrotic background measuring approximately 5 mm in the right lower limb.

A 70-year-old man with no relevant past or recent medical history, went to the emergency department due to disorientation, decreased muscle strength in the upper limbs and nonspecific chest pain with three days of evolution. Upon observation, he was apyretic and hemodynamically stable. Cardiopulmonary auscultation and abdominal examination revealed no alterations. In the neurological examination, we highlighted the temporal-spatial disorientation, proximal plegia of the upper limbs, paresis of the lower limbs and extended cutaneous-plantar reflex on the left side. We observed small linear subungual hemorrhages (Figure 1) and a lesion with necrotic background measuring approximately 5 mm in the distal portion of the right lower limb (Figure 2).

**Figure 3:**
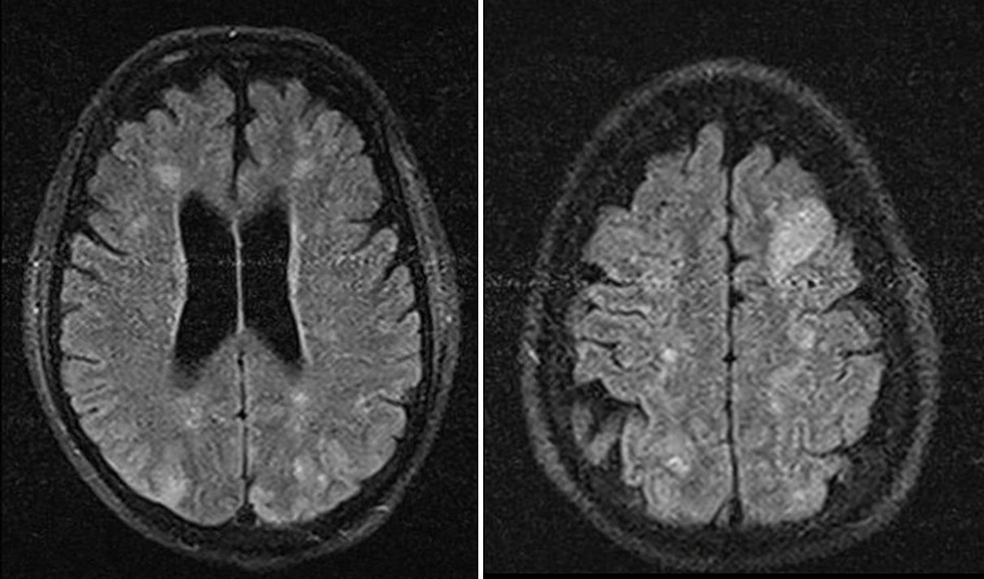
Multiple bilateral frontoparietal lesions, predominantly right, observed on brain magnetic resonance.

**Figure 4:**
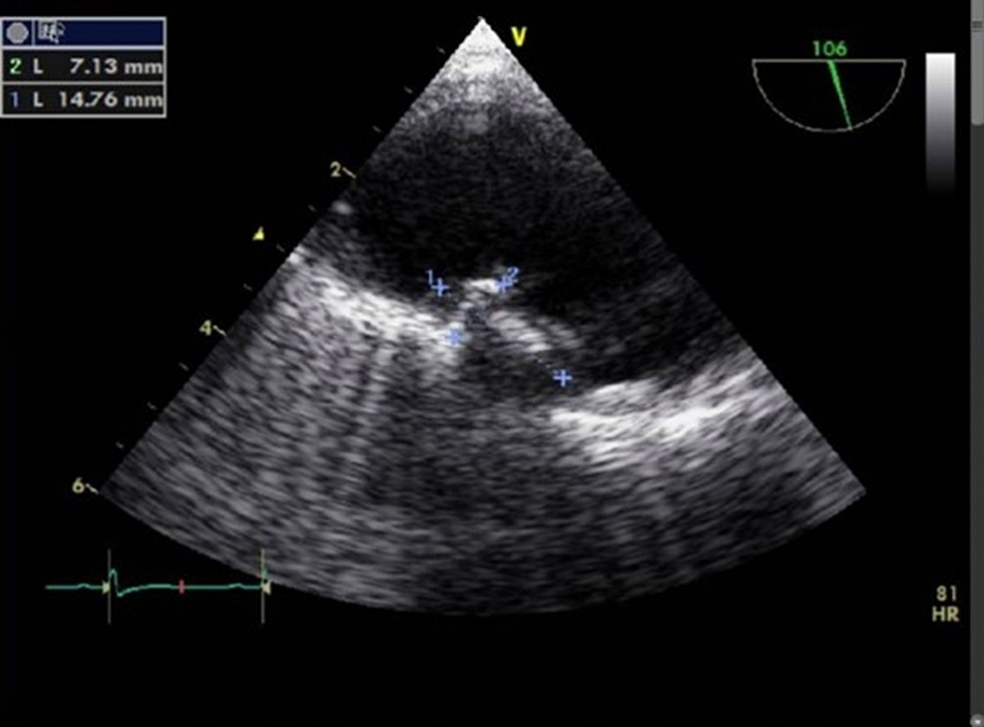
Thrombus in the aortic arch seen on transesophageal echocardiography.

Laboratory evaluation revealed 18,900 leukocytes/uL with eosinophilia of 9853/uL, and troponin elevation (7.2 ng/ml) with normal electrocardiogram. Cranioencephalic computed tomography (CT scan) showed ischemic lesions in several territories, better defined by brain magnetic resonance which documented multiple bilateral frontoparietal and right occipitotemporal lesions (Figure 3). Transesophageal echocardiography showed a mass measuring 7x14 mm in the arch of the aorta, compatible with a thrombus (Figure 4), confirmed by magnetic resonance imaging. The echocardiogram also showed left ventricular hypertrophy, with preserved systolic function and no restrictive pattern. Holter and echo-Doppler of carotid and vertebral arteries were normal.

In the presence of eosinophilia with organ damage, we started therapy with methylprednisolone 1 g/day for five days (followed by prednisolone 60 mg/day), anticoagulation with enoxaparin 1mg/kg twice daily and antiaggregation with acetylsalicylic acid 100 mg/day. Cardiac enzymes returned to normal levels and a gradual decline in eosinophils (up to 1200/uL after six weeks).

Other exams like abdominal ultrasound and body CT scan did not reveal abnormalities; skeletal radiography showed no calcified or lytic lesions; microbiological and parasitological tests were negative; viral serologies were negative; bone marrow analysis showed 18.6% eosinophils and no atypical cells; study of genetic mutations associated with clonal forms of eosinophilia was negative; the study of vasculitis and thrombophilia was negative (Table 1). As the investigation did not result in an etiology for eosinophilia, we admitted that it was an idiopathic form.

**Table 1:** Laboratory tests ANA - antinuclear antibodies; ANCA - antineutrophil cytoplasmic antibodies; BCR-ABL - breakpoint cluster region-abelson gene; CBFB - core-binding factor beta; CMV - cytomegalovirus; EBV - Epstein–Barr virus; HIV - human immunodeficiency virus; IgE - Immunoglobulin E; JAK2 - Janus kinase 2; PDGFRa - platelet-derived growth factor receptor, alpha polypeptide; PDGFRb - platelet-derived growth factor receptor, beta polypeptide.

Laboratory tests		Admission		Discharge	Reference values	
Hemoglobin (g/dl)		13.1		11.6	13-17	
Leukocytes /uL		18,900		7700	4000-11,000	
Neutrophils /uL		7400		4900	2100-7500	
Eosinophils /uL		9853		1200	0-500	
Lymphocytes /uL		960		1100	1000-4000	
Monocytes /uL		450		500	100-1000	
Basophils /uL		220		0	0-200	
Platelets /uL		179,000		272,000	150,000-400,000	
International Normalized Ratio		1.1			0.8-1.2	
D-dimers (ug/L)		1811			<500	
Creatinine (mg/dl)		1.15			0.7-1.3	
Urea (mg/dl)		53			<50	
Sodium (mEq/L)		140			135-147	
Potassium (mEq/L)		4.3			3.7-5.1	
Troponin T (ng/ml)		7.2		0.02	<0.03	
Creatinine kinases (U/L)		281			32-294	
Creatinine kinases - myocardial band (ng/ml)		48			<5	
Natriuretic peptide (pg/ml)		928			<100	
Aspartate aminotransferase (U/L)		29			<34	
Alanine aminotransferase (U/L)		25			<35	
gamma-glutamyl transpeptidase (U/L)		20			12-64	
Reactive C protein (mg/dl)		10.5			<0.5	
Thyroid Stimulating Hormone (mU/L)		1.87			0.4-4.5	
Free T4 (ng/dl)		1.4			0.7-1.8	
Vitamin B12 (pg/ml)		430			300-900	
Protein C, protein S and antithrombin III		Normal				
Lupus anticoagulant, anti-cardiolipin and anti-beta2 glycoprotein antibodies		Normal				
ANA, ANCA		Negative				
Peripheral blood smear		eosinophilia, without atypical cells				
Myelogram		18.6% eosinophils (16.6% mature), no myelodysplasia or atypical cells				
Genetic study		No mutations: PDGFRa, PDGFRb, BCR-ABL, CBFB, JAK2				
Protein Electrophoresis		No monoclonal pattern				
Total IgE (UI/ml)		433				<100
HIV, Hepatitis B and C		Negative				
Antibodies for Treponema pallidum		Negative				
Antibodies for CMV, EBV and herpes simplex		Negative				
Antibodies for Leptospira and Borrelia		Negative				
Feces	Microbiological exam	Negative				
	Parasitological exam	No protozoan cysts or Helminth eggs were observed				
Blood cultures		Negative				
Urine culture		Negative				

Despite corticosteroids therapy, anticoagulation and antiaggregation, the patient deteriorated with generalized hypertonia, new focal deficits - left central facial paresis, dysphagia and dysarthria, with evidence of new lesions on brain CT scan.

## Discussion

HES has an estimated prevalence between 0.36 to 6.3 per 100,000 and is more common in men in the proportion of 9:1 [1,2]. It is defined by persistent eosinophilia (peripheral blood eosinophil count greater than 1500/uL in at least two determinations [3,4]) and organ damage. It can be classified as: primary (clonal) relating to myeloid/lymphoid hematologic neoplasms and leukemias, with the proliferation of eosinophils and associating with mutations PDGFRa, PDGFRb, FGFR1, JAK2 or CBFB [5]; secondary (or reactive) that relates with allergic diseases, drug hypersensitivity reactions, infections, lymphomas, some solid tumors, connective tissue diseases, sarcoidosis, ulcerative colitis and vasculitis [5,6]; idiopathic - diagnosis of exclusion that corresponds to about 80% of cases [7]. Clinical presentation, as well as prognosis, is highly variable and depends on the underlying etiology and on the affected organs (most often the skin, heart, lungs, gastrointestinal tract and central nervous system [8]).

Eosinophils are granulocytic cells that contain active substances that directly exert their toxic effect, as well as cytokines that promote the activation of other cells and the inflammatory cascade. In the presence of hypereosinophilia, there is an unregulated release of these substances that cause damage by phenomena of inflammation, fibrosis, vasculitis and thrombosis [5].

Cardiovascular complications occur in 40 to 60% of cases [9] and are the major cause of morbidity and mortality [10]. A classic presentation of eosinophilic infiltration is Loeffler's endocarditis, in which there is thickening and fibrosis of the endocardium and consequent restrictive cardiomyopathy [11]. Cardiac involvement usually occurs in three phases: necrotic (acute, in which there is myocardial infiltration by eosinophils), thrombotic and fibrotic [10]. The clinical presentation can vary from heart failure, angor, arrhythmias, pericarditis and thrombotic phenomena (as we observed in this clinical case). Cardiac involvement can lead to the appearance of characteristic skin lesions named linear subungual hemorrhages (splinter hemorrhages), nail bed infarctions and ulcers, which usually occur in the necrotic phase [9,10]. Lab tests may show an increase in cardiac enzymes, d-dimers and type B natriuretic peptide. The electrocardiogram may be normal or show alterations in ventricular repolarization. The echocardiogram may reveal thrombi and alterations in cardiac function with a restrictive pattern [12,13]. Definitive diagnosis can be made by biopsy, but cardiac magnetic resonance is sensitive and non-invasive [10,14].

Neurological involvement is another important complication with an estimated incidence of stroke of 12% [15]. There are several mechanisms involved, the most important being embolization from a cardiac source [9]. Clinical presentation includes transient ischemic attacks and multifocal ischemic strokes (with additive deficits and high recurrence rates, even under anticoagulation and antiaggregation [9-12]), behavioral changes and confusional states (in the presence of encephalopathy caused by eosinophilic infiltration) and, more rarely, seizures and peripheral neuropathy [9].

The finding of potentially fatal HES complications is an indication for early treatment. Corticosteroids are one of the pillars of therapy [5]. Other therapeutic options, especially for patients refractory to corticosteroids, include hydroxyurea, interferon alpha and anti-interleukin 5. In the presence of cardiac thrombi, anticoagulation is indicated.

In this patient, despite the reduction in the number of eosinophils with therapy, there was a clinical worsening conditioned by thromboembolic phenomena, typically associated with a worse prognosis.

## Conclusions

This clinical case illustrates the complexity of this disease and its diagnostic process in an attempt to identify its etiology. The wide spectrum of presentations makes the diagnosis of HES a challenging one, and it must be remembered whenever eosinophilia and organ damage present in a patient. This pathology involves multidisciplinarity, with the internist being the patient's manager and articulating with other specialties such as Immunology, Infectiology, Cardiology and Neurology.
